# Erroneous treatment of syphilis with benzyl penicillin in an era with benzathine benzylpenicillin shortages

**DOI:** 10.1136/sextrans-2019-054380

**Published:** 2020-01-23

**Authors:** Silvia Nieuwenburg, Noor Rietbergen, Danielle van Zuylen, Clarissa Vergunst, Henry de Vries

**Affiliations:** 1 Department of Infectious Diseases, Public Health Service, Amsterdam, The Netherlands; 2 General Practitioners Practice Keizersgracht, Amsterdam, Noord-Holland, The Netherlands; 3 Department of Dermatology, Meibergdreef 9, Amsterdam Institute for Infection and Immunity (AI&II), Amsterdam UMC, University of Amsterdam, Amsterdam, The Netherlands

**Keywords:** syphilis, penicillin, therapy

The WHO estimates there are 5.6 million new cases of syphilis annually.^1^ The recommended choice of treatment for syphilis is 2.4 million units of benzathine benzylpenicillin (BBP), also called benzathine penicillin G, with no documented risk of antibiotic resistance. In 2015, the WHO began to receive country reports of BBP stock-outs.^2^ As with many off-patent drugs, the price competition of BBP is stiff. As a result, many manufacturers have discontinued production, and the stock-out risk has increased.^3^ From 2015 onwards, the Netherlands has been confronted with BBP stock-outs.

At the STI clinic in Amsterdam, we were recently confronted with a treatment failure in a patient with syphilis who was treated with benzylpenicillin (BP) intramuscular injections by his general practitioner (GP). In a second case, we were consulted by a GP who considered a treatment with BP. In both cases, the GP was advised by the pharmacist to administer intramuscular BP as an alternative treatment for syphilis. Also, the national pharmacist information system provided confusing advices on the alternatives for BBP treatment.

BP is a short-acting antibiotic, usually administered intravenously for the treatment of neurosyphilis and congenital syphilis in daily dosage regimes for multiple days. BBP is a salt form of BP stabilised with the benzathine group. When administered intramuscularly, BBP is slowly hydrolysed into BP and thus works as a long-acting depot penicillin.

BBP successfully cures uncomplicated early syphilis infections (the clinical stages 1 and 2 and the early latent stage) with a single dose of 2.4 million units intramuscular. For the late latent and unknown duration syphilis stages, weekly 2.4 million units intramuscular for 3 weeks is the recommended regime. Under no circumstances can intramuscular BP replace intramuscular BBP following the same dosage regimes for the treatment of uncomplicated syphilis.

Duration of treponemicidal level of antimicrobials should be at least 7–10 days to cover a number of division times (30–33 hours).^4^ Extended treatment is needed as the duration of infection increases (more relapses have been seen in later stages after short courses of treatment), possibly because of more slowly dividing treponemes in late syphilis. It is for this reason that BP with an elimination half-life of 15–30 min after intramuscular injection does not suffice. Due to the benzathine stabiliser, BBP induces prolonged low concentrations of BP over 2–4 weeks after a single intramuscular dose. Therefore, BBP remains the mainstay for the treatment of uncomplicated syphilis.

In the absence of BBP, doxycycline 100 mg two times per day for 14 days can be used as an alternative for the treatment of early uncomplicated syphilis and 100 mg two times per day for 28 days for the late latent stage and stage of unknown duration syphilis. Strict serological follow-up is recommended when treating with doxycycline to exclude treatment failure. Although the names are quite similar, physicians and pharmacists need to be aware of the differences in the working mechanism of BP and BBP to avoid using inappropriate treatments of infectious syphilis.
